# Supplementary material for: Effects of motor imagery training on skeletal muscle contractile properties in sports science students
Source: PeerJ. 2022 Nov 24;10:e14412. doi: 10.7717/peerj.14412 (PMC9701499; doi:10.7717/peerj.14412)
Supplement: Table S1 — Means and standard deviations (in brackets) for the following tensiomyographic parameters: radial displacement (Dm), contraction velocity (Vc), and delay time (Td) separated into effort condition and the three intervention groups (MI = motor imagery, PE = physical exercise, VI = visual imagery). [file peerj-10-14412-s002.docx]

|  | | MI (*n* = 13) | | | PE (*n* = 14) | | | VI (*n* = 15) | | |  |
| --- | --- | --- | --- | --- | --- | --- | --- | --- | --- | --- | --- |
|  |  | Pre | Post | | Pre | Post | | Pre | Post | |  |
| Dm (mm) | 0 N | 15.05 (± 3.1) | | 14.18 (± 3.1) | 15.72 (± 3.4) | | 15.22 (± 2.6) | 14.01 (± 3.2) | | 14.52 (± 1.7) | |
|  | 50 N | 1.92 (± 0.8) | | 1.82 (± 0.8) | 2.34 (± 1.2) | | 2.38 (± 0.8) | 2.03 (± 0.9) | | 2.13 (± 0.6) | |
| V_c_ (mm/ms) | 0 N | 0.438 (± 0.09) | | 0.409 (± 0.11) | 0.455 (± 0.11) | | 0.449 (± 0.09) | 0.441 (± 0.08) | | 0.432 (± 0.07) | |
|  | 50 N | 0.042 (± 0.02) | | 0.041 (± 0.02) | 0.047 (± 0.03) | | 0.046 (± 0.02) | 0.037 (± 0.01) | | 0.042 (± 0.01) | |
| T_d_ (ms) | 0 N | 28.65 (± 4.4) | | 27.45 (± 3.7) | 28.18 (± 2.5) | | 27.23 (± 2.5) | 29.85 (± 5.5) | | 29.34 (± 5.2) | |
|  | 50 N | 24.82 (± 2.9) | | 25.06 (± 1.7) | 25.88 (± 2.3) | | 25.09 (± 2.1) | 25.46 (± 2.5) | | 25.42 (± 2.9) | |
